# Semen quality and metabolic profile in people with type 1 diabetes with and without erectile dysfunction: a cross-sectional study

**DOI:** 10.1007/s40618-023-02285-z

**Published:** 2024-01-16

**Authors:** M. Longo, P. Caruso, C. Varro, M. Tomasuolo, P. Cirillo, L. Scappaticcio, L. Romano, D. Arcaniolo, M. I. Maiorino, G. Bellastella, M. De Sio, K. Esposito

**Affiliations:** 1 Department of Advanced Medical and Surgical Sciences, University of Campania “Luigi Vanvitelli”, Piazza Luigi Miraglia 2, 80138 Naples, Italy; 2 Division of Endocrinology and Metabolic Diseases, University of Campania “Luigi Vanvitelli”, Naples, Italy; 3 Unit of Urology, Department of Neurosciences, Reproductive Sciences, and Odontostomatology, University of Naples “Federico II”, Naples, Italy; 4 Department of Woman, Child and General and Specialized Surgery, University of Campania ‘Luigi Vanvitelli’, Naples, Italy

**Keywords:** Type 1 diabetes, CGM, Semen quality, ED, Glucose control, BIA

## Abstract

**Purpose:**

The aim of the present study is to evaluate the association of metabolic and glycemic variables with semen parameters in patients with type 1 diabetes (T1D) with and without erectile dysfunction (ED).

**Methods:**

The study population included 88 adults with T1D using a continuous glucose monitoring, of whom 28 with ED (ED group) and 60 without it (NO ED group). All men completed the International Index of Erectile Function (IIEF-5) and underwent body composition analysis (BIA) and semen analysis.

**Results:**

ED group showed worse HbA1c levels [median (IQR), 8.4 (7.7, 9.9) vs 7.4 (7, 8.2) %, *P* < 0.001)], higher insulin dose [60 (51, 65) vs 45 (38, 56) UI/die, *P* = 0.004)] and a higher total body water and intracellular water as compared with ED group. Men in the ED group presented higher semen volume [2.8 (2.6, 4.2) vs 2.5 (2.2, 2.7) mL, *P* < 0.001] and sperm concentration [24 (19, 29) vs 20 (12, 23) mil/mL, *P* = 0.010], but reduced sperm progressive motility [28 (25, 35) vs 35 (25, 36) %, *P* = 0.011], higher rate of non-progressive motility [15 (10, 15) vs 10 (5, 10) %, *P* < 0.001] and higher rate of typical morphology [7(5, 8) vs 5 (4, 5) %, *P* = 0.001]. Based on multivariate logistic regression analysis performed to assess the association between clinical variables and ED, intracellular water (OR 3.829, 95% CI 1.205, 12.163, *P* = 0.023) resulted as the only independent predictor of ED.

**Conclusion:**

Men with T1D and ED showed worse metabolic profile which is associated with poor semen quality, as compared with those without ED.

**Supplementary Information:**

The online version contains supplementary material available at 10.1007/s40618-023-02285-z.

## Introduction

Type 1 diabetes is an autoimmune metabolic disease characterized by chronic hyperglycemia and high glucose fluctuations due to absolute insulin deficiency. The number of incident and prevalent cases of type 1 diabetes is increasing worldwide every year [1]; specifically, in 2021, around 1,211,900 children and adolescents under 20 years were estimated to have type 1 diabetes worldwide, with the highest incidence rates in Northern Europe and the lowest in China [2].

In recent years, the use of continuous glucose monitoring devices (CGM) has become widespread worldwide. In the United States, their use among people with type 1 diabetes has increased approximately from 7% in 2011 to 28% in 2017 [3]. CGM measures interstitial glucose levels every 5 min, allowing the collection of a large number of glucose readings aggregated in metrics that can be used to assess glucose control in clinical practice, including percentage of time in normoglycemia (TIR), in hyperglycaemia (TAR), in hypoglycaemia (TBR), the glucose management indicator (GMI) and the coefficient of variation (CV) [4]. Interestingly, high glycaemic variability and low TIR have recently been associated with micro- and macrovascular complications in both type 1 and type 2 diabetes [5].

Diabetes has been associated with erectile dysfunction (ED), the incidence of which is 3.5 times higher in diabetic men than in non-diabetic individuals [6]. The pathogenesis of ED in diabetes is multifactorial and includes both organic and psychological factors. Overweight and obesity, smoking, hypertension, dyslipidemia, hypogonadism, unhealthy diet and physical inactivity are important risk factors for ED in men with diabetes [7]. Conversely, the role of glycaemic control, including glucose fluctuations, in the development of ED and premature ejaculation (PE) in men with type 1 diabetes is still uncertain [8, 9].

Both diabetes and erectile disorders may affect male fertility. A higher rate of ED has been observed among men with infertility compared with fertile men [10]. Evidence coming from small cross-sectional studies investigating semen parameters in diabetic patients revealed reduced sperm motility in subjects with type 1 diabetes as compared to controls, with contradictory results regarding other semen parameters [11].

To our knowledge, the contribution of the metabolic outlook to the quality of semen is not well known in men with type 1 diabetes. Moreover, the relationship between type 1 diabetes, ED and semen parameters has not been so far investigated. Therefore, the aim of this study is to evaluate the association of both metabolic and glycemic variables with the principal semen parameters in patients with type 1 diabetes with and without ED.

## Materials and methods

### Patients

This is an exploratory analysis from the observational METRO study. The METRO study is a single-center, longitudinal, observational study aimed at evaluating the metabolic and endocrinological profile of young subjects with type 1 diabetes in transition from Pediatric clinic to the adult Diabetes center at the Teaching hospital of University of Campania “Luigi Vanvitelli” [12]. From June 2021 to October 2022, men included in the METRO study were consecutively screened for this cross-sectional analysis if they: (1) were aged ≥ 18 and ≤ 35 years, (2) were on stable and optimized intensive insulin treatment, including multiple daily insulin injection (MDI) and continuous subcutaneous insulin infusion (CSII), with an individual dietary plan; (3) were using real-time CGM (Dexcom G6) or iCGM (Free Style, Abbott) for at least 6 months prior the study, (4) had a sensor use > 70% and were sharing data on web-based platform (Libreview or Clarity), (5) had stable couple relationship or sexual activity (masturbation) in the previous month, and (6) did not use phosphodiesterase type 5 inhibitors (PDE5-i). Exclusion criteria were considered the presence of any uncontrolled chronic diseases not including diabetes complications (neoplasms, severe neurodegenerative diseases, major depression or other psychiatric disorders, penis disorders, drug or alcohol abuse), presence of mono- and/or bilateral varicocoele, overt hypogonadism and compensated forms characterized by total testosterone ≥ 10.5 nmol/L and luteinizing hormone > 9.4 U/L, hyperprolactinemia, cryptorchidism, the use of drugs associated with adverse effects on erectile function, a history of urological surgery, lower urinary tract symptoms, and pelvic trauma in the last 6 months. Informed consent was obtained from every subject. The manuscript has been prepared in accordance with Strengthening the Reporting of Observational Studies in Epidemiology recommendations (STROBE, Supplementary Table S1).

### Anthropometric measures and laboratory analyses

The height and weight of each participant were measured using a Seca 200 scale (Seca, Hamburg, Germany) with an annexed stadiometer. Body mass index (BMI) was calculated as weight (in kilograms) divided by height (expressed in meters squared). Waist circumference was also measured. Arterial blood pressure was measured three times while subjects were sitting after 15 min resting. Assays for fasting glucose, total cholesterol, low-density (LDL) and high-density (HDL) lipoprotein cholesterol, triglyceride levels, HbA1c, and testosterone were performed in the hospital’s chemistry laboratory. Fasting glucose was measured using glucose oxidase method and glycated hemoglobin (HbA1c) was measured using high-pressure liquid chromatography. Serum total cholesterol (TC), triacylglycerols (TG), HDL cholesterol (HDL-C), and LDL cholesterol (LDL-C) levels were measured using enzymatic methods performed on clinical chemistry analyzer. Testosterone, FSH, LH were measured by radioimmunoassay.

### Bioelectrical impedance analysis (BIA)

For each patient, we evaluated body composition with bioelectrical impedance analysis (multi-frequency BIA, Human Im Touch, DS Medica Srl; Milan, Italy). BIA is a sample, safe, inexpensive and non-invasive method to estimate body composition. BIA analyzers inject an alternating sinusoidal electric current at different frequencies through active electrodes and measures bioelectrical impedance (z) and phase angle (Φ) of human body [13]. In biological systems, electrical conduction is related to water and ionic distribution in the conductor, resulting far greater in fat-free mass (FFM) than fat mass (FM) [14]. We used the two-compartment model evaluating FM and FFM in addition to the following parameters: intracellular water (ICW), total body water/fat-free mass (TBW/FFM), body cell mass (BCM) and basal metabolic rate (BMR). All participants were supine with limbs slightly spread apart from the body, refrained from eating, drinking, and exercising for 6 h before testing. Subjects were tested in the supine position with arms and legs abducted from the body.

### Assessment of sexual function

All participants in the study were asked to complete the Italian version of two validated multiple-choice self-reported questionnaires assessing both erectile and ejaculatory functions. Questionnaires were sent by email to participants in the study by a dedicated web-based platform (www.surveyspace.it). Erectile function was investigated by completing the abbreviated form of the International Index of Erectile Function (IIEF-5) [15], which comprises items 2, 4, 5, 7, and 15 of the full scale IIEF-15 and assures simplicity and immediacy in its compilation. According to the recommended scoring system, a total score of 21 or less indicates the presence of ED. Furthermore, we used the five-item premature ejaculation diagnostic tool (PEDT) [16] to assess and investigate the control of ejaculatory function, the frequency of PE, the minimum sexual stimulation, and both distress and interpersonal difficulty. A score of 8 or lower excluded a diagnosis of PE.

### Nutritional assessment

Adherence to Mediterranean diet was assessed using a validated 14-item PREDIMED (PREvención con DIeta MEDiterránea) questionnaire [17]. For each item was assigned score 1 and 0 and PREDIMED score was calculated as follows: 0–5, lowest adherence; 6–9, average adherence; score ≥ 10, highest adherence [17]. Furthermore, to evaluate additional lifestyle-factors beyond diet (sociability, sleep and rest and conviviality), we used the MEDLIFE (MEDiterranean LIFEstyle) questionnaire, a 28-item questionnaire divided into 3 blocks: 1) Mediterranean food consumption (15 items); 2) Mediterranean dietary habits (7 items); 3) Physical Activity, rest, social habits and conviviality (6 items). Each answer was scored as 0 when not meeting the cutoff established for the item or 1 when meeting the specific cutoff for the corresponding item, so that the complete MEDLIFE ranged from 0 to 28, with a higher value indicative of greater adherence to Mediterranean lifestyle [18].

### Semen analysis

Semen samples were obtained by masturbation in the privacy room adjacent to the laboratory, after 3–5 days of abstinence [19]. After liquefaction, semen was analyzed according to the sixth edition of the WHO Laboratory Manual for the Examination and Processing of Human Semen 2021 [20]. The samples were maintained at 37 °C until assay. The ejaculate volume was estimated in calibrated tubes. pH was assessed by using pH indicator strips (LLG Labware, Lab Logistics Group, Meckenheim Germany). For the assessment of sperm motility, 10-µl of well-mixed semen was placed on a glass slide with a coverslip. The preparation was immediately examined under 10× magnification with a microscope. Sperm motility was classified as progressive or non-progressive. Sperm concentration was estimated in duplicate and the average was calculated. Sperm morphology was assessed by smearing 10 μL semen according to the WHO method, fixing in etherethanol and staining with Papanicolaou and mounting in Eukitt for long-term storage of the samples. Subjects were classified as “not normozoospermic” if at least one among the main sperm parameters were altered (i.e. sperm concentration, total sperm number, progressive and total motility and sperm morphology) or “normozoospermic”, if all semen parameters were above the calculated 5th centile of the subjects used for the WHO decision limits [21]. All samples were estimated by the same operator.

### Assessment of CGM-related metrics

CGM-related metrics of the 14 days before the visit were collected by the above-mentioned web-based platforms and analyzed by displaying the ambulatory glucose profile (AGP). They included the coefficient of variation (CV), the glucose management indicator (GMI), the percentage of time spent in the range of normoglycemia (TIR, 70–180 mg/dL), the percentage of time spent below range (TBR, level 1 between 54 and 69 mg/dL, level 2 < 54 mg/dL), the percentage of time spent above range (TAR, level 1 between 181 and 250 mg/dL, level 2 between 251 and 400 mg/dL).

### Statistical analysis

Data in tables and figures concerning normally distributed variables are presented as mean ± SD, while non-normally distributed continuous variables are presented as median and interquartile ranges. Differences between groups were evaluated by the two-sided Student’s t-test or Wilcoxon–Mann–Whitney test. The χ^2^-test was used to compare dichotomous variables. Spearman’s or Pearson’s correlation coefficients were used to test the associations between different variables. Multivariable logistic regression models were performed to test the contribution of independent variables (factors exhibiting significant correlation) to the dependent variable (ED or not). After assessing collinearity bias by calculating the variance inflation factors, not normally distributed data have been entered in the model as dichotomous variables, calculating for each variable values above or below the median. P value lower than 0.05 was considered statistically significant. All statistical analyses were performed using SPSS software.

## Results

One-hundred twenty-seven patients were screened for eligibility and 25 men were excluded (18 for hypogonadism; 7 for PDE5 inhibitors use; Supplementary Figure S1). Fourteen out of the remaining 102 men refused to complete the IIEF-5 questionnaire. A total of 88 men with type 1 diabetes were included in the study, of whom 28 with erectile dysfunction (ED group) and 60 without ED (NO ED group). Mean age was 25.8 years and mean diabetes duration was 15.3 years. Mean BMI was 24 kg/m^2^ and 36% of patients were overweight. Eight patients (10%) had microvascular complications and 20 (23%) had autoimmune diseases associated. Moreover, 20% of participants in the study used a real-time CGM and 80% an iCGM device. The clinical and metabolic characteristics of the population divided according to the presence of ED are illustrated in Table 1. The two groups were well balanced for age, diabetes duration, weight, BMI, blood pressure, autoimmune diseases and microvascular complications. Patients of ED group showed worse glycemic control [ED group vs NO ED group, median (IQR), HbA1c 8.4 (7.7, 9.9) vs 7.4 (7, 8.2) %, *P* < 0.001)] and assumed higher insulin dose [60 (51, 65) vs 45 (38, 56) UI/die, *P* = 0.004)] than men of NO ED group. A higher proportion of patients on lipid-lowering therapy was found in ED group (42%) compared with that found in NO ED group (3%, *P* < 0.001). No differences on CGM-derived metrics of glucose control were observed between men with ED and those without ED, except for sensor time activity [82 (76, 90) vs 93 (80, 100)%, *P* < 0.001)] and TAR level 1 [22 (21, 29) vs 26 (23, 31)%, *P* = 0.012]. Prevalence of PE was higher in ED group compared to NO ED group (45 vs 0%, *P* < 0.001). The percentage of fatherhood was not different in ED and NO ED groups, 5% and 6%, respectively.

**Table 1:** Characteristics of type 1 diabetic men with and without erectile dysfunction (ED)

Parameters	ED (*N* = 28)	NO ED (*N* = 60)	P
Age, years	26 ± 6	26 ± 4	0.654
Diabetes duration, years	14 (12, 17)	15 (12, 22)	0.106
Smokers, n (%)	12 (43)	36 (60)	0.203
Weight, kg	77 (64, 78)	73 (66, 81)	0.248
BMI, kg/m^2^	25.9 (21.4, 27.3)	23.8 (21.3, 26.5)	0.066
Overweight, *n* (%)	16 (57)	16 (27)	0.011
WC, cm	90 (75, 93)	82 (78, 84)	0.364
SBP, mmHg	120 (110, 125)	120 (110, 130)	0.082
DBP, mmHg	80 (70, 80)	80 (70, 90)	0.931
FG, mg/dL	199 (184, 235)	200 (130, 230)	0.578
HbA1c, %	8.4 (7.7, 9.9)	7.4 (7.0, 8.2)	< 0.001
Testosterone, ng/mL	6.3 (4.9, 8.0)	6.5 (5.0, 7.9)	0.730
LH, UI/L	2.1 (1.1, 2.4)	2.2 (0.9, 2.5)	0.258
FSH, UI/L	2.5 (2.1, 3.2)	2.1 (0.8, 3.1)	0.346
Total daily insulin dose, UI/day	60 (51, 65)	45 (38, 56)	0.004
CSII users, *n* (%)	3 (10)	9 (15)	0.745
Lipid-lowering therapy, *n* (%)	12 (42)	2 (3)	< 0.001
Autoimmune diseases, *n* (%)	8 (29)	12 (20)	0.535
Microvascular complications, *n* (%)	3 (10)	5 (8)	0.712
PE prevalence, *n* (%)	12 (43)	0 (0)	< 0.001
*CGM-related metrics*			
Time sensor activity, %	82 (76, 90)	93 (80, 100)	< 0.001
TIR, % (70–180 mg/dL)	56.6 (46, 68)	54 (46, 64)	0.531
TAR level 1, % (181–250 mg/dL)	22 (21, 29)	26 (23, 31)	0.012
TAR level 2, % (251–400 mg/dL)	12 (8, 22)	15 (6, 20)	0.363
TBR level 1, % (54–69 mg/dL)	2 (1, 8)	4 (1, 6)	0.835
TBR level 2, % (< 54 mg/dL)	0 (0, 3)	1 (0, 1)	0.288
CV, %	43.9 (33.5, 45.9)	38.7 (34.6, 45.1)	0.778
GMI, %	7.2 (6.9, 7.8)	7.3 (6.9, 7.7)	0.808

Data are expressed as mean and standard deviation or median and interquartile range or number and percentage. BMI: body mass index; CV: coefficient of variation; DBP: diastolic blood pressure; FG: fasting glucose; FSH: follicle-stimulating hormone; GMI: glucose management indicator; LH: luteinizing hormone; PE: premature ejaculation; SBP: systolic blood pressure; TAR: time above range; TBR: time below range; TIR: time in range; WC: waist circumference

**Fig. 1:**
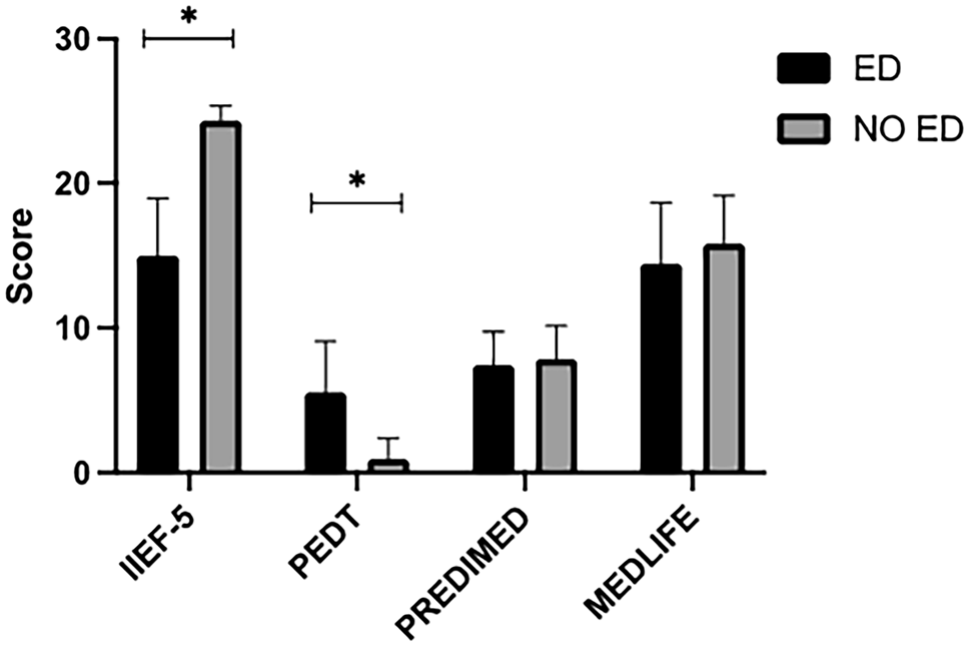
Score for sexual function (IIEF-5 and PEDT) and adherence to Mediterranean diet (PREDIMED and MEDLIFE) in patients with type 1 diabetes with and without erectile dysfunction. *P < 0.001

As expected, ED group showed a significantly lower IIEF-5 score [17 (12, 18) vs 25 (23, 25), *P* < 0.001)] and higher PEDT score [7 (2, 9) vs 0 (0, 1), *P* < 0.001)] as compared with NO ED group (Fig. 1). On the other hand, patients of the two groups did not differ for PREDIMED and MEDLIFE score.

Participants in ED group showed higher TBW/FFM [77.7 (69.4, 89) vs 70 (67.4, 77.5) %, *P* = 0.048] and ICW [29.9 (26.4, 32.1) vs 28.2 (25, 29.8) L, *P* = 0.021], as compared with NO ED group (Table 2). There were no other differences in other parameters of BIA.

**Table 2:** Body composition parameters of type 1 diabetic men with and without erectile dysfunction (ED)

Parameters	ED (*n* = 28)	NO ED (*n* = 60)	*P*
TBW/FFM, %	77.7 (69.4, 89.0)	70.0 (67.4, 77.5)	0.048
ICW, L	29.9 (26.4, 32.1)	28.2 (25.0, 29.8)	0.021
FM, %	13.8 (6.0, 26.0)	11.1 (7.0, 21.4)	0.235
FFM, %	86.2 (74.0, 89.0)	88.0 (76.0, 92.7)	0.108
BCM, Kg	33.6 (29.9, 38.8)	34.8 (31.0, 37.1)	0.554
BMR, kcal/die	1700 (1665, 1823)	1763 (1675, 1785)	0.756

Data are expressed as median and interquartile range

BCM: body cell mass; BMR: basal metabolic rate; FFM: fat-free mass; FM: fat mass; ICW: intracellular water; TBW/FFM: total body water/fat-free mass

Table 3 depicts the semen parameters in the two groups. Patients of ED group reported higher semen volume [2.8 (2.6, 4.2) vs 2.5 (2.2, 2.7) mL, *P* < 0.001], higher sperm concentration [24 (19, 29) vs 20 (12, 23) mil/mL, *P* = 0.010] and total count [70 (49, 101) vs 47 (30, 58) mil/mL, *P* = 0.005] as compared with NO ED group. Moreover, patients with ED had reduced percentage of sperm progressive motility [28 (25, 35) vs 35 (25, 36) %, *P* = 0.011], associated with higher non-progressive motility [15 (10, 15) vs 10 (5, 10) %, *P* < 0.001] and higher sperm typical morphology [7(5, 8) vs 5 (4, 5) %, *P* = 0.001].

**Table 3:** Semen parameters in type 1 diabetic men with and without erectile dysfunction (ED)

Parameters	ED (*n* = 28)	NO ED (*n* = 60)	*P*
Semen volume, mL	2.8 (2.6, 4.2)	2.5 (2.2, 2.7)	< 0.001
pH	8 (7.9, 8.5)	8.0 (7.8, 8.5)	0.197
Sperm concentration, mil/mL	24 (19, 29)	20 (12, 23)	0.010
Sperm total count, mil/ejaculate	70 (49, 101)	47 (30, 58)	0.005
Sperm progressive motility, %	28 (25, 35)	35 (25, 36)	0.011
Sperm non-progressive motility, %	15 (10, 15)	10 (5, 10)	< 0.001
Sperm total motility, %	45 (40, 45)	45 (35, 46)	0.525
Sperm absent motility, %	55 (55, 60)	55 (54, 65)	0.645
Sperm typical morphology, %	7 (5, 8)	5 (4, 5)	0.001
Testicular volume, mL	17 (16, 20)	17 (15, 19)	0.356

Data are expressed as median and interquartile range

Data on the population stratified in normozoospermic and not normozoospermic subjects are illustrated in Supplementary Table S2. Patients with normozoospermia showed better glycemic control [HbA1c 7.2 (7, 8) vs 8.3 (7.6, 9.7) %, *P* < 0.001], associated with lower CV [35.7 (34.1, 43.9) vs 42.4 (35.7, 46) %, *P* = 0.023], lower TAR2 [8.5 (6, 19) vs 17 (7.5, 23) %, *P* = 0.037] and higher TIR [61.5 (49, 64) vs 52 (44, 64.5) %, *P* = 0.023] than men with no normozoospermia. Moreover, a higher proportion of smokers (*P* = 0.022) and autoimmune diseases (*P* < 0.001) were found in people without normozoospermia that those with normozoospermia.

Results on the study population after excluding subjects with BMI > 25 kg/m^2^ are illustrated in Supplementary Table S3 and S4. Patients of ED group reported higher percentage of non-progressive motility [12 (10, 15) vs 10 (9, 10) %, *P* = 0.009] and higher sperm typical morphology [6 (4, 7) vs 5 (5, 6) %, *P* = 0.002] as compared to those without ED. No difference was observed on BIA parameters between the two groups.

Results from univariate analysis are reported in Table 4. In the overall population, IIEF-5 score was negatively associated with HbA1c (*r* = − 0.405, *P* < 0.001), total daily insulin dose (*r* = − 0.391, *P* < 0.001), ICW (*r* = − 0.257, *P* = 0.016), semen volume (*r* = − 0.394, *P* < 0.001), sperm concentration (*r* = − 0.485, *P* < 0.001), sperm total count (*r* = − 0.500, *P* < 0.001) and sperm typical morphology (*r* = − 0.373, *P* < 0.001). No significant association was found between TBW/FFM and sperm progressive motility with IIEF-5 score.

**Table 4:** Statistical associations between metabolic and semen parameters and IIEF-5 score by univariate analysis

Parameters	*r*_sp_	*P*
HbA1c, %	− 0.405	< 0.001
Total daily insulin dose, UI per day	− 0.391	< 0.001
TBW/FFM, %	− 0.217	0.420
ICW, L	− 0.257	0.016
Volume, mL	− 0.394	< 0.001
Sperm concentration, mil/mL	− 0.485	< 0.001
Sperm total count, mil/ejaculate	− 0.500	< 0.001
Sperm progressive motility, %	− 0.039	0.718
Sperm typical morphology, %	− 0.373	< 0.001

ICW: intracellular water; TBW/FFM: total body water/fat-free mass

Based on multivariate logistic regression analysis performed to assess the association between clinical variables and ED, ICW (OR 3.829, 95% CI 1.205, 12.163, *P* = 0.023) resulted as the only independent predictor of ED (Table 5).

**Table 5:** Statistical associations between metabolic and semen parameters and ED by multiple logistic regression analysis

Parameters	Odds ratio	95% CI		*P*
HbA1c, %	1.772	0.511	6.145	0.367
Total daily insulin dose, UI/day	2.060	0.622	8.782	0.255
ICW, L	3.829	1.205	12.163	0.023
Sperm concentration, mil/mL	2.507	0.657	9.571	0.179
Normozoospermia	2.982	0.842	10.565	0.090

Dependent Variable: IIEF-5

ED: erectile dysfunction; ICW: intracellular water

## Discussion

To the best of our knowledge, this is the first study to report the relationship between metabolic and CGM-derived metrics on semen parameters in patients with type 1 diabetes with and without ED. At similar lifestyle habits, worsen HbA1c levels and higher total body water and intracellular water were found in subjects with ED compared to those without ED. In addition, patients with type 1 diabetes and ED had higher semen volume and sperm concentration associated with a higher rate of typical morphology, lower progressive motility and higher non-progressive motility than in the NO ED group. Furthermore, intracellular water resulted as independent predictor of erectile dysfunction.

The effects of glycemic control and hyperglycemia in the pathogenesis of ED are still unclear. Some observational studies have shown an association between poor glycemic control, expressed by elevated HbA1c levels, and ED [22–24], while other studies have found no association [25]. On the other hand, contrary to what has been observed in women with type 1 diabetes [26], glucose variability does not seem to play a crucial role for ED, as a study of 112 young male patients with type 1 diabetes found no difference in the prevalence of male sexual dysfunction in those with high and low glucose variability [8]. Our results are consistent with these data, as patients with ED had poorer long-term glucose control (HbA1c), but no difference in glycemic metrics measured over a short period of time.

Diabetes may affect sperm parameters, as men with type 1 diabetes have poorer sperm quality compared to healthy, age-matched controls [27]. In a cross-sectional analysis of 32 men with type 1 diabetes and 20 fertile controls, diabetic men were found to have lower progressive sperm motility, although sperm concentration did not differ between groups [28]. In addition, fasting hyperglycemia was found to be an independent predictor of lower progressive motility [27]. Significantly worse sperm parameters, including motility, were found in patients with type 1 diabetes with poor metabolic control and neuropathy [29]. Interestingly, in some patients with diabetes, a significant increase in sperm concentration and total sperm count was associated with lower sperm motility and volume [30]. Moreover, in a Japanese cross-sectional study of 564 newlywed men, subjects with poor semen quality showed higher age, BMI, and higher fasting blood glucose [31]; erectile function resulted as an independent predictor for poor semen, confirming the association between the two variables in non-diabetic men. Furthermore, in a recent longitudinal study of 12 patients undergoing obesity surgery, sexual function improved after 18 months of follow-up, without effects on semen parameters even after a significant weight loss (from 42.37 ± 4.44 to 29.6 ± 3.77 kg/m^2^) [32].

There is evidence that the severity of ED increases with worsening semen quality and is more severe in men with abnormal sperm parameters than in men with lesser abnormalities or healthy men [33]. However, there is currently a lack of data on sperm parameters in diabetic men with and without ED. In our study, subjects with type 1 diabetes and ED had higher semen volume and sperm concentration, but lower progressive sperm motility, a higher rate of non-progressive motility and a higher rate of typical morphology, with similar testosterone levels. Long-term chronic hyperglycemia exacerbates the body's oxidative stress response and vascular endothelial dysfunction, which can lead to structural abnormalities of the testes and epididymis and consequently of the spermatozoa [30]. Furthermore, increased endothelial dysfunction and oxidative stress seem to mediate the development of ED in diabetic patients [34].

Oxidative stress and overproduction of ROS may result in mitochondrial dysfunction and in axonemal damage, which lead to reduced sperm motility. Indeed, there is also evidence that in healthy men the lower motility of spermatozoa has been associated with a higher oxidative stress in seminal plasma and increased levels of inflammatory cytokines [35].

In addition, ED status and associated psychological and organic disorders can also affect male fertility. Indeed, ejaculation frequency is also an important factor that influences semen parameters. The changes in sperm parameters observed in our study could be due to the lower frequency of sexual intercourse of the subjects with ED in the last month, although all subjects reported abstinence of 3–5 days at the time of the study. It is widely admitted that prolonged sexual abstinence may be beneficial for semen volume and sperm concentration; however, lack of ejaculation also displays adverse consequences on sperm motility and viability, which may explicate the reduced motility in our patients [36]. We can hypothesize that the reduction in sexual activity may determine a retention of semen with a prolongation of sperm living in the testes, experiencing reduction in motility and higher semen volume, with no effects on sperm morphology. Indeed, although an increased rate of typical morphology has been observed in ED group compared to ED group, both cohorts of patients presented values included in normal range.

A lot of cardiovascular risk factors may have an independent role on the risk of ED, including hypertension, smoking, weight and dyslipidemia. In our study, ED group showed a higher proportion of patients on lipid lowering treatments. There is evidence that ED seems to be more related to the underlying disease than to its specific therapy [37]; our findings confirm that participants with ED had a worse glyco-metabolic control, needing lifelong treatments, compared with those without ED.

Moreover, higher body weight and BMI have been associated with a higher risk of ED [38]. Although in our study, the proportion of overweight individuals in the ED group was higher than in the NO ED group, we found no difference in fat mass and free fat mass between the two groups on BIA. Conversely, the men with ED had higher intracellular and total body water content despite a similar lifestyle to the men without ED. However, in a cross-sectional study of 4108 haemodialysis patients, a significant inverse correlation was found between hydration status and IIEF levels [39]. These data may suggest that hydration status influences sexual function and contributes to the development of ED through worsening inflammatory status in type 1 diabetes. The reasons for this association remain unknown.

The main limitations of this study are its cross-sectional nature that does not allow to make inference regarding cause and effect, the exploratory nature of the study without predetermined calculation of sample size. Semen analysis was performed once, adding some spontaneous variability of the semen quality. Moreover, free testosterone levels were not reported; however, our populations included young adults with fair glucose control and a small proportion of overweigh/obese men. Given the epidemiological nature, the potential for residual confounding by uncontrolled covariates is possible. Major strengths include the young age of participants in the study, the relatively large number of men involved (*n* = 88), the possibility to discriminate between men with ED and without ED, the use of validated tool for the evaluation of dietary habits, the contemporary evaluation of semen parameters and body composition.

In conclusion, subjects with type 1 diabetes and ED showed worse metabolic profile in terms of long-term glucose control and body composition, which is associated with lower sperm progressive motility and higher non-progressive motility in semen than diabetic men without ED. However, these findings should be interpreted with caution, due to the small sample size. Further studies should investigate whether these changes in semen quality could affect fertility in large cohorts of patients with type 1 diabetes.

### Supplementary Information

Below is the link to the electronic supplementary material.

Supplementary file1 (DOCX 71 KB)

## Data Availability

All data generated or analysed during this study are included in this published article [and its supplementary information files].
