# Supplementary material for: Semen quality and metabolic profile in people with type 1 diabetes with and without erectile dysfunction: a cross-sectional study
Source: J Endocrinol Invest. 2024 Jan 16;47(7):1787–95. doi: 10.1007/s40618-023-02285-z (PMC11196288; doi:10.1007/s40618-023-02285-z)
Supplement: Supplementary file 1 — Supplementary file1 (DOCX 71 KB) [file 40618_2023_2285_MOESM1_ESM.docx]

**Semen quality and metabolic profile in people with type 1 diabetes with and without erectile dysfunction: a cross-sectional study**

**Supplementary file**

Supplementary Table S1 ……………………………………………………………………………………….. pag. 2

Supplementary Table S2 ……………………………………………………………………………………….. pag. 6

Supplementary Table S3 ……………………………………………………………………………………….. pag. 7

Supplementary Table S4 ……………………………………………………………………………………….. pag. 8

Supplementary Figure S1………………………………………………………………………………………. pag. 9

**Supplementary Table S1**. STROBE Statement—checklist of items that should be included in reports of observational studies

|  | Item No | Recommendation | Page n. | Relevant text from manuscript |
| --- | --- | --- | --- | --- |
| **Title and abstract** | 1 | (*a*) Indicate the study’s design with a commonly used term in the title or the abstract | 1,2 | Title and abstract |
|  |  | (*b*) Provide in the abstract an informative and balanced summary of what was done and what was found | 2 | Abstract |
| Introduction | | |  |  |
| Background/rationale | 2 | Explain the scientific background and rationale for the investigation being reported | 3,4 | Introduction |
| Objectives | 3 | State specific objectives, including any prespecified hypotheses | 4 | Introduction |
| Methods | | |  |  |
| Study design | 4 | Present key elements of study design early in the paper | 4 | Materials and methods |
| Setting | 5 | Describe the setting, locations, and relevant dates, including periods of recruitment, exposure, follow-up, and data collection | 4,5 | Materials and methods |
| Participants | 6 | (*a*) *Cohort study*—Give the eligibility criteria, and the sources and methods of selection of participants. Describe methods of follow-up  *Case-control study*—Give the eligibility criteria, and the sources and methods of case ascertainment and control selection. Give the rationale for the choice of cases and controls  *Cross-sectional study*—Give the eligibility criteria, and the sources and methods of selection of participants | 4 | Materials and methods |
|  |  | (*b*) *Cohort study*—For matched studies, give matching criteria and number of exposed and unexposed  *Case-control study*—For matched studies, give matching criteria and the number of controls per case |  |  |
| Variables | 7 | Clearly define all outcomes, exposures, predictors, potential confounders, and effect modifiers. Give diagnostic criteria, if applicable | 4 | Materials and methods |
| Data sources/ measurement | 8* | For each variable of interest, give sources of data and details of methods of assessment (measurement). Describe comparability of assessment methods if there is more than one group | *5,6,7,8* | Materials and methods |
| Bias | 9 | Describe any efforts to address potential sources of bias | Not applicable |  |
| Study size | 10 | Explain how the study size was arrived at | Not applicable |  |
| Quantitative variables | 11 | Explain how quantitative variables were handled in the analyses. If applicable, describe which groupings were chosen and why | 8 | Statistical analysis |
| Statistical methods | 12 | (*a*) Describe all statistical methods, including those used to control for confounding | 8 | Statistical analysis |
|  |  | (*b*) Describe any methods used to examine subgroups and interactions | 8 | Statistical analysis |
|  |  | (*c*) Explain how missing data were addressed | 8 | Statistical analysis |
|  |  | (*d*) *Cohort study*—If applicable, explain how loss to follow-up was addressed  *Case-control study*—If applicable, explain how matching of cases and controls was addressed  *Cross-sectional study*—If applicable, describe analytical methods taking account of sampling strategy | Not applicable |  |
|  |  | (*e*) Describe any sensitivity analyses | Not applicable |  |

| Results | | |  | |  | |  |
| --- | --- | --- | --- | --- | --- | --- | --- |
| Participants | 13* | (a) Report numbers of individuals at each stage of study—eg numbers potentially eligible, examined for eligibility, confirmed eligible, included in the study, completing follow-up, and analysed | | 8 | | Results, Supplementary Figure S1 | |
|  |  | (b) Give reasons for non-participation at each stage | | Supplementary file pag 7 | | Supplementary Figure S1 | |
|  |  | (c) Consider use of a flow diagram | | Supplementary file pag 7 | | Supplementary Figure S1 | |
| Descriptive data | 14* | (a) Give characteristics of study participants (eg demographic, clinical, social) and information on exposures and potential confounders | | 8 | | Results | |
|  |  | (b) Indicate number of participants with missing data for each variable of interest | | 8 | | Results | |
|  |  | (c) *Cohort study*—Summarise follow-up time (eg, average and total amount) | |  | |  | |
| Outcome data | 15* | *Cohort study*—Report numbers of outcome events or summary measures over time | |  | |  | |
|  |  | *Case-control study—*Report numbers in each exposure category, or summary measures of exposure | |  | |  | |
|  |  | *Cross-sectional study—*Report numbers of outcome events or summary measures | | *8* | | *Results* | |
| Main results | 16 | (*a*) Give unadjusted estimates and, if applicable, confounder-adjusted estimates and their precision (eg, 95% confidence interval). Make clear which confounders were adjusted for and why they were included | | Not applicable | |  | |
|  |  | (*b*) Report category boundaries when continuous variables were categorized | | 8 | | Results | |
|  |  | (*c*) If relevant, consider translating estimates of relative risk into absolute risk for a meaningful time period | | Not applicable | |  | |
| Other analyses | 17 | Report other analyses done—eg analyses of subgroups and interactions, and sensitivity analyses | | Not applicable | |  | |
| Discussion | | |  | |  | |  |
| Key results | 18 | Summarise key results with reference to study objectives | | 10 | | Discussion | |
| Limitations | 19 | Discuss limitations of the study, taking into account sources of potential bias or imprecision. Discuss both direction and magnitude of any potential bias | | 13 | | Discussion | |
| Interpretation | 20 | Give a cautious overall interpretation of results considering objectives, limitations, multiplicity of analyses, results from similar studies, and other relevant evidence | | 11,12,13 | | Discussion | |
| Generalisability | 21 | Discuss the generalisability (external validity) of the study results | | 13 | | Discussion | |
| Other information | | |  | |  | |  |
| Funding | 22 | Give the source of funding and the role of the funders for the present study and, if applicable, for the original study on which the present article is based | | 13 | | Funding section | |

*Give information separately for cases and controls in case-control studies and, if applicable, for exposed and unexposed groups in cohort and cross-sectional studies.

**Note:** An Explanation and Elaboration article discusses each checklist item and gives methodological background and published examples of transparent reporting. The STROBE checklist is best used in conjunction with this article (freely available on the Web sites of PLoS Medicine at http://www.plosmedicine.org/, Annals of Internal Medicine at http://www.annals.org/, and Epidemiology at http://www.epidem.com/). Information on the STROBE Initiative is available at www.strobe-statement.org.

**Supplementary Table S2.** Characteristics of type 1 diabetic men normozoospermic and not normozoospermic.

| **PARAMETERS** | **Normozoospermic subjects (N = 40)** | **Not normozoospermic subjects (N = 60)** | **P** |
| --- | --- | --- | --- |
| Age, years | 26.5 (24, 30) | 23.5 (21, 27) | < 0.001 |
| Diabetes duration, years | 15 (12, 23) | 14.5 (12, 17.5) | 0.421 |
| Smokers, n (%) | 16 (40) | 32 (67) | 0.022 |
| Weight, Kg | 75 (64, 81.2) | 73.2 (66.8, 77.8) | 0.690 |
| BMI, Kg/m^2^ | 23.8 (20.4, 26) | 24 (22.4, 26.6) | 0.081 |
| Overweight, n (%) | 12 (30) | 20 (42) | 0.363 |
| WC, cm | 81.3 (78, 91) | 82.5 (78.5, 89.5) | 0.423 |
| SBP, mmHg | 123 (120, 130) | 115 (110, 125) | 0.005 |
| DBP, mmHg | 77.5 (70, 90) | 80 (70, 80) | 0.838 |
| FG, mg/dL | 196 (184, 230) | 199.5 (146.5, 223.5) | 0.594 |
| HbA1c, % | 7.2 (7, 8) | 8.3 (7.6, 9.7) | <0.001 |
| Testosterone, ng/mL | 6.4 (5, 8.0) | 6.3 (4.9, 8.1) | 0.841 |
| LH, UI/L | 2.3 (1.2, 2.4) | 2.1 (1, 2.3) | 0.379 |
| FSH, UI/L | 2.4 (1.8, 3.3) | 2.2 (1.2, 3) | 0.290 |
| Total daily insulin dose, UI/day | 53.3 (38, 65) | 53 (41.3, 57.5) | 0.594 |
| CSII users, n (%) | 7 (17) | 5 (10) | 0.514 |
| Lipid-lowering therapy, n (%) | 4 (10) | 8 (17) | 0.552 |
| Autoimmune diseases, n (%) | 0 (0) | 20 (42) | <0.001 |
| Microvascular complications, n (%) | 2 (5) | 6 (12) | 0.283 |
| PE prevalence, n (%) | 4 (10) | 8 (17) | 0.552 |
| *CGM-related metrics* |  |  |  |
| Time sensor activity, % | 93 (79, 94) | 85 (79.5, 99) | 0.950 |
| TIR, % (70-180 mg/dL) | 61.5 (49, 64) | 52 (44, 64.5) | 0.023 |
| TAR level 1, % (181-250 mg/dL) | 24.5 (23, 30) | 24 (21, 30.5) | 0.347 |
| TAR level 2, % (251-400 mg/dL) | 8.5 (6, 19) | 17 (7.5, 23) | 0.037 |
| TBR level 1, % (54-69 mg/dL) | 3 (0, 5) | 4 (1, 6.5) | 0.079 |
| TBR level 2, % (< 54 mg/dL) | 0 (0, 1) | 1 (0, 1.5) | <0.001 |
| CV, % | 35.7 (34.1, 43.9) | 42.4 (35.7, 46) | 0.023 |
| GMI, % | 7.2 (7.1, 7.7) | 7.6 (6.9, 7.8) | 0.251 |
| Data are expressed as mean and standard deviation or median and interquartile range or number and percentage. BMI, body mass index; CV, coefficient of variation; DBP, diastolic blood pressure; FG, fasting glucose; FSH, follicle-stimulating hormone; GMI, glucose management indicator; LH, luteinizing hormone; PE, premature ejaculation; SBP, systolic blood pressure; TAR, time above range; TBR, time below range; TIR, time in range; WC, waist circumference. | | | |

**Supplementary Table S3.** Semen parameters in type 1 diabetic men with and without erectile dysfunction (ED), after excluding those with BMI > 25 Kg/m^2^.

| **Parameters** | **ED (n = 12)** | **NO ED (n = 44)** | **P** |
| --- | --- | --- | --- |
| Semen volume, mL | 2.6 (2.5, 2.9) | 2.5 (2.2, 2.8) | 0.196 |
| pH | 8 (7.9, 8.5) | 8 (7.8, 8.2) | 0.337 |
| Sperm concentration, mil/mL | 21 (19, 28) | 20 (13, 21) | 0.053 |
| Sperm total count, mil/eiaculate | 52 (49, 70) | 47 (31, 58) | 0.080 |
| Sperm progressive motility, % | 30 (25, 35) | 35 (25, 36) | 0.134 |
| Sperm non-progressive motility, % | 12 (10, 15) | 10 (9, 10) | 0.009 |
| Sperm total motility, % | 45 (35, 45) | 45 (35, 46) | 0.495 |
| Sperm absent motility, % | 55 (55, 65) | 55 (55, 65) | 0.734 |
| Sperm typical morphology, % | 6 (4, 7) | 5 (5, 6) | 0.002 |
| Testicular volume, mL | 18.5 (16, 20) | 17 (15, 18) | 0.023 |

Note: data are expressed as median and interquartile range.

**Supplementary Table S4.** Body composition parameters of type 1 diabetic men with and without erectile dysfunction (ED), after excluding those with BMI > 25 Kg/m^2^.

| **Parameters** | **ED (n = 12)** | **NO ED (n = 44)** | **P** |
| --- | --- | --- | --- |
| TBW/FFM, % | 69.4 (63.6, 90) | 69 (67.4, 74.3) | 0.638 |
| ICW, L | 29.2 (26.4, 32.1) | 28.5 (28.1, 30) | 0.542 |
| FM, % | 9.2 (5.7, 13.8) | 10 (5.6, 19.9) | 0.468 |
| FFM, % | 88.8 (86.2, 94.3) | 88.9 (78.6, 94.4) | 0.881 |
| BCM, Kg | 33.6 (29.9, 39.2) | 35.3 (34.3, 37.6) | 0.429 |
| BMR, kcal/die | 1823 (1673, 1920) | 1763 (1675, 1841) | 0.152 |
| Note: data are expressed as median and interquartile range.  Abbreviations: BCM, body cell mass; BMR, basal metabolic rate; FFM, fat-free mass; FM, fat mass; ICW, intracellular water; TBW/FFM, total body water/fat-free mass. | | | |

**Supplementary Figure S1.** Process of patients selection.


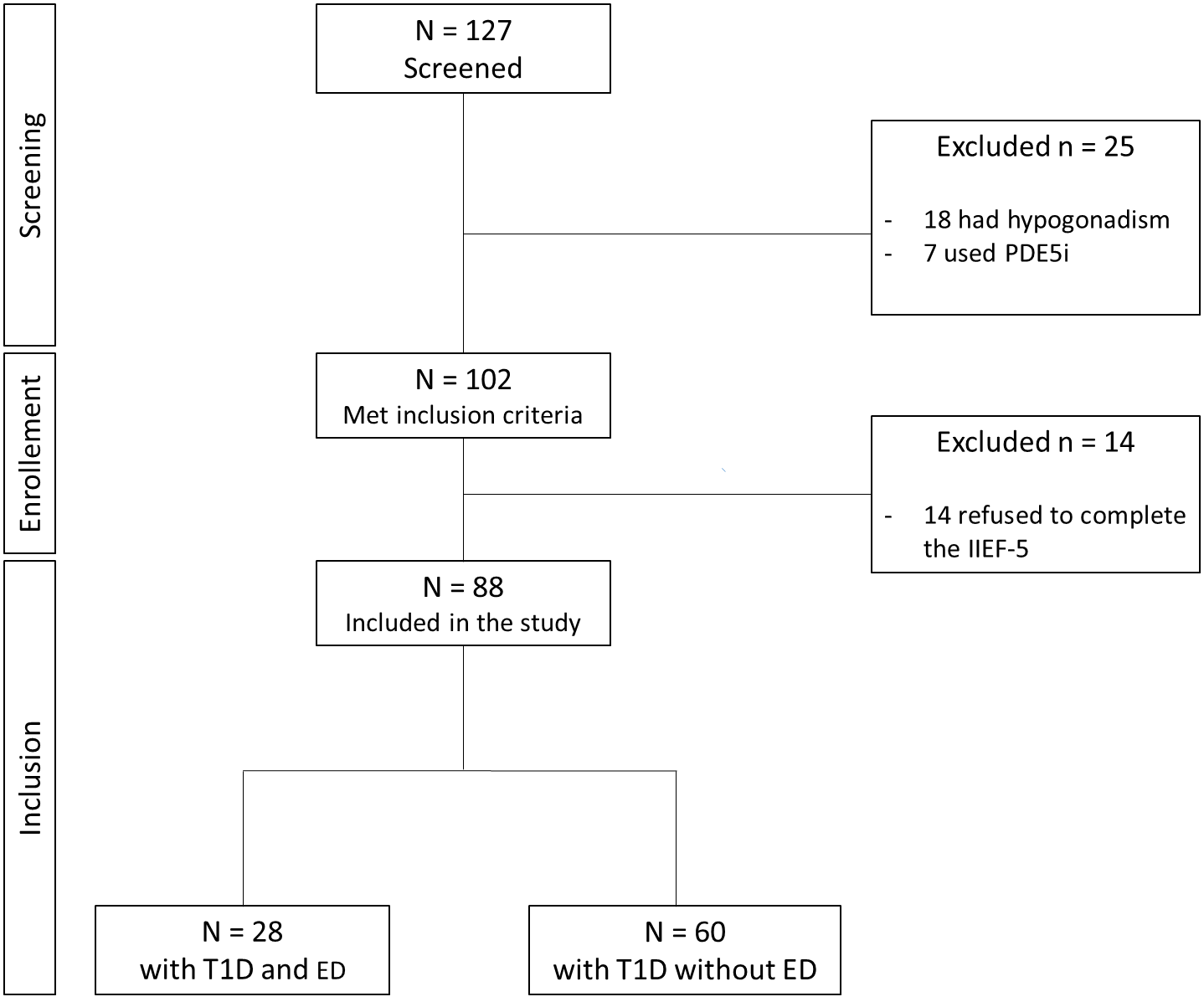


ED, erectile dysfunction; IIEF-5, international index of erectile function; PDE5i, PDE5 inhibitors.
